# COMT Val^158^Met Genotype Selectively Alters Prefrontal [^18^F]Fallypride Displacement and Subjective Feelings of Stress in Response to a Psychosocial Stress Challenge

**DOI:** 10.1371/journal.pone.0065662

**Published:** 2013-06-14

**Authors:** Dennis Hernaus, Dina Collip, Johan Lataster, Jenny Ceccarini, Gunther Kenis, Linda Booij, Jens Pruessner, Koen Van Laere, Ruud van Winkel, Jim van Os, Inez Myin-Germeys

**Affiliations:** 1 Department of Psychiatry and Neuropsychology, South Limburg Mental Health Research and Teaching Network, EURON, School for Mental Health and NeuroScience MHeNS Maastricht University, Maastricht, The Netherlands; 2 Faculty of Psychology, Open University of The Netherlands, Heerlen, The Netherlands; 3 Nuclear Medicine Division, University Hospital and Catholic University Leuven, Leuven, Belgium; 4 Sainte-Justine Hospital Research center, Montreal, Quebec, Canada; 5 Department of Psychiatry, University of Montreal, Montreal, Quebec, Canada; 6 Department of Psychiatry, McGill University, Montreal, Quebec, Canada; 7 Douglas Mental Health Institute, Department of Psychiatry, McGill University, Montreal, Quebec, Canada; 8 University Psychiatric Centre Catholic University Leuven, Kortenberg, Belgium; 9 King’s College London, King’s Health Partners, Department of Psychosis Studies, Institute of Psychiatry, London, United Kingdom; Centre for Addiction and Mental Health, Canada

## Abstract

Catechol-O-methyltransferase (COMT) plays an essential role in degradation of extracellular dopamine in prefrontal regions of the brain. Although a polymorphism in this gene, COMT Val^158^Met, affects human behavior in response to stress little is known about its effect on dopaminergic activity associated with the human stress response, which may be of interest for stress-related psychiatric disorders such as psychosis. We aimed to investigate the effect of variations in COMT genotype on in vivo measures of stress-induced prefrontal cortex (PFC) dopaminergic processing and subjective stress responses. A combined sample of healthy controls and healthy first-degree relatives of psychosis patients (n = 26) were subjected to an [^18^F]fallypride Positron Emission Tomography scan. Psychosocial stress during the scan was induced using the Montreal Imaging Stress Task and subjective stress was assessed every 12 minutes. Parametric t-maps, generated using the linear extension of the simplified reference region model, revealed an effect of COMT genotype on the spatial extent of [^18^F]fallypride displacement. Detected effects of exposure to psychosocial stress were unilateral and remained restricted to the left superior and right inferior frontal gyrus, with Met-hetero- and homozygotes showing less [^18^F]fallypride displacement than Val-homozygotes. Additionally, Met-hetero- and homozygotes experienced larger subjective stress responses than Val-homozygotes. The direction of the effects remained the same when the data was analyzed separately for controls and first-degree relatives. The human stress response may be mediated in part by COMT-dependent dopaminergic PFC activity, providing speculation for the neurobiology underlying COMT-dependent differences in human behaviour following stress. Implications of these results for stress-related psychopathology and models of dopaminergic functioning are discussed.

## Introduction

Catechol-O-methyltransferase (COMT) plays an essential role in degradation of extracellular dopamine in prefrontal regions of the mammalian brain, where dopamine levels are relatively low [1]. COMT’s influence on cortical dopamine levels has been ascribed to low cortical expression of dopamine transporter (DAT), leaving the neurotransmitter susceptible to the enzymatic activity of COMT [2]. A polymorphism in the COMT gene, Val^158^Met, affects enzymatic activity: Met-hetero- and homozygotes tend to have a higher cortical dopaminergic tone, due to lower enzymatic activity of COMT, than Val-homozygotes [3], [4].

It has been shown that the COMT Val^158^Met polymorphism influences dopaminergic prefrontal cortex (PFC) functions such as working memory [5]. The effects of COMT genotype on PFC dopaminergic functioning, however, seem pleiotropic: while some PFC functions may benefit from a specific COMT genotype, other functions may not [6]. For instance, transgenic mice overexpressing the human COMT-Val polymorphism performed worse on a working memory task than Val-knockout mice, but showed a marked resistance to stress [7]. In humans, similar results have been reported as Val-homozygotes appear more stress-resistant [8], [9], [10], while carriers of the Met-allele perform better on tasks indexing cognition [3], [5]. These results converge on the idea of a trade-off between stress-sensitivity and cognitive ability; whereas Met-allele loading may increase cognitive performance at the cost of increased stress-sensitivity, Val-allele loading may generate stress-resistance in combination with suboptimal cognitive performance [6], [7].

Studies examining COMT-dependent brain activity associated with cognitive processes consistently revealed more efficient dorsolateral PFC activity for Met-allele carriers compared to Val-homozygotes [3], [5]. However, no studies to date have investigated COMT-dependent differences in brain dopamine levels in response to stress. As it has been proposed that the human stress response may be mediated by dopaminergic signaling [11], [12], it may be hypothesized that there is an effect of COMT on the PFC dopaminergic response to stress, influencing stress-sensitivity at the behavioral level [13]. Given the fact that COMT genotype effects on dopaminergic PFC activity may be task-dependent [3], [6], the incorporation of a valid stress challenge is a crucial element in elucidating COMT-dependent differences in dopaminergic PFC activity in response to stress. The need for experimental paradigms, compared to basal conditions, to elucidate between-group (e.g. genotype-based) differences in dopaminergic activity has been confirmed in animal studies [14] and has been speculated to be the case in human genetics research [15], suggesting that differences in dopamine activity only become apparent under demanding circumstances, for example of cognitive or emotional nature.

Decreased levels of PFC dopamine have been hypothesized to be an important feature of psychiatric disorders, in particular psychosis [11], [16]. Observed differences in cognitive performance [17] and differences in tolerance to stress [18], [19] between individuals suffering from psychotic symptoms and controls may be underlain by differences in available PFC dopamine. It is therefore that investigating whether the dopaminergic stress response is COMT-dependent may be relevant for psychosis and other stress-related disorders.

The present study aimed to assess the effect of COMT on stress-induced PFC dopamine signaling, as measured by [^18^F]fallypride positron emission tomography (PET), thereby attempting to elucidate for the first time COMT-dependent differences in the dopaminergic stress response. To investigate this hypothesis, a sample previously described by Lataster and colleagues [11] was used, consisting of both healthy controls and healthy first-degree relatives of psychosis patients. [^18^F]Fallypride is a high-affinity and selective dopamine D_2/3_ radiotracer, which has been used to investigate striatal and extrastriatal D_2/3_ availability [20], [21], even in brain regions such as the PFC where D_2/3_ receptor density is an order of magnitude lower than in the striatal regions [22]. To detect and map PFC dopamine release during a stress task, we used the linearized simplified reference region method (LSRRM) [23], a method that makes use of the endogenous neurotransmitter competition with the radioligand at the receptor sites; hereby the spatial extent of the estimated stress-induced ligand displacement by the stimulus is thought to be a proxy of increased dopamine release [11], [24], [25], [26], [27]. The LSRRM permits the detection of voxel-wise transient changes, with the advantage of additionally investigating regions with low radiotracer uptake such as the PFC [26]. The primary goal of this work was to examine the effect of COMT genotype on PFC [^18^F]fallypride displacement in response to stress. Secondly, we also investigated the effect of COMT genotype on subjective stress responses. Our hypotheses were investigated in a combined sample of healthy controls and healthy first-degree relatives of psychosis patients [21].

## Methods

### Ethical Approval

The standing medical ethics committee of Maastricht University approved the study. Participants signed informed consent before taking part. The study was carried out according to the principles of the Declaration of Helsinki.

### Sample

The sample consisted of 14 first-degree relatives of patients with psychotic disorder and 12 controls (not at increased risk of psychosis). The effects of psychosocial stress on cortical dopaminergic activity in this sample have been previously described [11], [21] and for the purposes of this manuscript were genotyped for the COMT Val^158^Met polymorphism. Inclusion criteria were (i) age 18–65 years; (ii) sufficient command of the Dutch language to understand instructions. Exclusion criteria were (i) intellectual impairment; (ii) head trauma with loss of consciousness (>5 min)/central neurological disorder; (iii) endocrine disorder; (iv) cardiovascular disorder; (v) diagnosis of psychiatric illness according to the criteria of the Diagnostic and Statistical Manual of Mental Disorders (DSM-IV-TR) (The American Psychiatric Association, 2000) (generated using the Operational Criteria Checklist (OPCRIT) program [28]) (vi) history of psychotropic medication and/or substance abuse; (vii) current/previous abuse of illicit drugs; (viii) >5 standard units of alcohol per day or a history of alcohol abuse; (ix) metal elements in the body; (x) claustrophobia; (xi) pregnancy or lactation.

### Psychosocial Stress Challenge

The psychosocial stress task used in the present study, the Montreal Imaging Stress Task (MIST) [12], a PET adapted version of the Trier Mental Challenge Task, has been described in detail in other reports [11], [12] and has successfully been used to elicit psychosocial stress and changes in dopaminergic activity. Moreover, changes in [^18^F]fallypride displacement induced by the MIST are positively correlated with the subjective stress response [11], [21]. The experiment consisted of a control and experimental condition administered consecutively during a single-day PET scan protocol. During the control condition, participants performed 6-minute blocks of mental arithmetic on a computer screen without time constraints or performance feedback. In the stress condition, participants performed similar mental arithmetic as in the control condition, with the addition of information about the total number of errors, expected average number of errors, time spent on current problem, a tone rising in frequency indicating the end of the response interval and evaluative feedback of a confederate investigator. In order to validate that the task elicited stress and assess subjective levels of stress in response to the task [11] a 7-point Likert scale questionnaire consisting of the following questions: “I feel relaxed”, “I’m in control”, “I feel comfortable among these people”, “I feel judged by these people”, and “I do not live up to expectations” (1 = not at all, 7 = very) was administered to the participants every 12 min. An extensive debriefing session took place at the end of the experiment, in which participants were told that the task was specifically designed to be out of reach of their mental capacity.

### Positron Emission Tomography

#### 1. PET acquisition and data analysis

**Figure 1:**
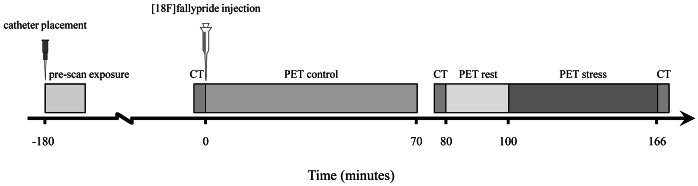
Graphical overview of the scanning protocol. Prior to [^18^F]fallypride injection into the antecubital vein at t = 0, a CT scan was obtained. After injection a 70-minute control block was followed by a 5-minute break. Then, a second CT scan was obtained, followed by a brief block of rest. The stress condition started at t = 80 and scanning ended at approximately t = 160, followed by a third and final CT scan.

[^18^F]Fallypride has reliably been used to detect task-related extrastriatal dopamine release [11], [20], [21], [26], [29]. Participants received 183.2 MBq (SD = 7.6) of [^18^F]fallypride (for radiotracer preparation: Text S1) in a slow intravenous bolus injection through a catheter in the antecubital vein. Simultaneously upon radiotracer injection, dynamic emission scans were initiated in three-dimensional mode using a HiRez Biograph 16 PET/computed tomography (CT) camera (Siemens Medical Solutions, Inc.). Emission data were collected in two segments, one during the control condition (86 min) and one during the stress condition of the MIST (100 min). There was a brief break of 10 min between the control and experimental segment, after which subjects were, if necessary, repositioned on the scanner bed and the second PET emission data were collected for another 86 min in total. In order to ensure that “activation” (i.e. presence or absence of additional dopamine release, reflected by changes in ligand displacement) was not a result of repositioning or the simple act of getting up from the scanner, a low-dose (80 kV tube potential, 11 mA·s) CT scan was obtained to later correct for movement throughout the paradigm. A CT scan was conducted at the beginning of each PET segment (immediately before tracer injection and at 80 min post injection) and at the end of emission scan. No task was presented during the first 20 min of this second emission segment. This, additionally, minimized risk of carry-over effects from the control condition into the stress condition, and maximized psychological impact of the stressor. A graphical timeline of the scanning protocol is provided in Fig. 1. Images were reconstructed using a 3D ordered-subset expectation maximization (OSEM) iterative reconstruction including model-based scatter and attenuation correction based on a measured attenuation map acquired by the CT, with a final spatial resolution of 4 mm. For each participant, a T1-weighted and standard transverse T2 brain magnetic resonance image (MRI; 1.5 Tesla Vision Scanner, Siemens, Germany) was obtained. Parameters for the T1 3D Magnetization Prepared Rapid Acquisition Gradient Echo sequence were: TR = 0 ms, TE = 4 ms, flip angle = 12°, inversion time 300 ms, matrix 256×256, 160 sagittal contiguous slices of 1 mm.

Realignment, coregistration to MRI, normalization (T1-weighted Montreal Neuroimaging Inistitute (MNI) template) and smoothing (4-mm full width half maximum (FWHM)) were performed before applying the kinetic model using SPM8 (Statistical Parametric Mapping, The Wellcome Department of Cognitive Neurology, London, UK). Briefly, for each subject, the dynamic reconstructed images were first realigned using a rigid transformation to correct for potential effects of head movement, then co-registered to the corresponding MRI scan, and finally normalized to a specific T1-weighted MNI template.

The cerebellum was used as a reference region for [^18^F]fallypride representing a cerebral area with a paucity of dopamine D_2/3_ receptors [30]. Two binary masks were created based on the corresponding normalized MRI, using an inhouse created set of volumes-of-interest (VOI) and the Talairach atlas (Talairach & Tournoux, 1988) for each participant. One binary mask image was created containing all regions of interest in the PFC (BA8 = posterior superior frontal gyrus; BA9/BA46 = dorsolateral prefrontal cortex; BA10 = ventromedial prefrontal cortex; BA11 = medial orbitofrontal cortex; BA44 = inferior frontal gyrus, pars opercularis; BA45 = inferior frontal gyrus, pars triangularis; and BA47 = inferior frontal gyrus, pars orbitalis, BA = Brodmann Area), and a second mask contained only the cerebellum. The mask used for analysis contained an average total volume of 26841 voxels. The regions were based on the anatomical delineation of the human PFC, in line with others studies using a similar approach [11], [26], [31].

#### 2. The linearized simplified reference region model

For each participant, VOI-based analyses were performed by estimating the kinetic parameters using the linear simplified reference region model (LSRRM) [23] and the PET time-activity curves (TACs).

The LSRRM is based on a single scanning session with a baseline and an activation condition and accounts for time-dependent changes in ligand displacement, assuming that the steady state is not maintained during the activation condition, and has been successfully implemented in studies investigating stimulus-related dopaminergic processing [11], [24], [26], [27], [29]. The LSRRM yields statistical parametric voxel-wise t-maps of the “activation” parameter γ (*t* = γ/sd(γ), where sd(γ) is the standard deviation parametric value for γ. Subsequently, the spatial extent of the estimated stress-induced ligand displacement was presented as percentage of voxels within a given VOI exceeding a threshold *t*>4.5, which corresponds to a p<.000002 one-tailed t-test comparing increased ligand displacement vs. the control state, or a false discovery rate (FDR)-corrected p<.05 t-test (.05/average total number of voxels analyzed per subject ( = about 24913) [11], [26], [27], [29]. To sum up, the LSRRM allows voxel-wise comparisons to investigate tracer displacement in response to a stimulus using γ (*t* = γ/sd(γ), with voxels in which t exceeds the FDR-corrected significance threshold revealing [^18^F]fallypride displacement [25]. This outcome measure is interpreted as a “difference score”, reflecting task- or stimulus-related tracer displacement. The amount of active voxels per VOI is averaged per group (in this case, COMT genotype) and a linear regression analysis is performed to investigate if the amount of active voxels differs per group.

The LSRRM, in addition to voxel-wise statistics, also yields additional, standard, parameters such as binding potential relative to non-displaceable radioligand (BP_ND)_) [32], K2 and K2a. BP_ND_ in this case describes the complete scanning paradigm (i.e. no ΔBP_ND_) and was calculated according to Alpert and colleagues’ definition [23] (BP_ND_ is calculated as (K2/K2a)−1). More information about the kinetic model used in the study can be found in Text S2.

### COMT Val^158^Met Genotyping

Genomic deoxyribonucleic acid (DNA) was collected from blood. DNA was isolated manually according to the Promega protocol or with the Autogenflex3000. The COMT Val^158^Met polymorphism (rs4680) was genotyped using a TaqMan®SNP Genotyping assay (assay ID C__25746809_50, Applied Biosystems, Nieuwerkerk a/d IJssel, The Netherlands). The assay was run on a 7900HT Fast Real-Time PCR System (Applied Biosystems).

### Analyses

To investigate the association between stress-induced prefrontal dopamine release and COMT genotype in a combined sample of healthy volunteers and healthy first-degree relatives of psychosis patients, VOI-based (see section “1. PET acquisition and data analysis”) linear regression analyses were conducted using task-induced changes in [^18^F]fallypride displacement as dependent variable (quantified as the percentage of voxels exceeding FDR-corrected significance threshold of p(α(FDR) = 5%) <.05). COMT Val^158^Met was used as a dichotomous categorical variable, with Val-homozygotes as one group and Met-hetero- and homozygotes as the other. Rationales for grouping of Met-allele carriers were: i) grouping of Met-allele carriers has been done before in genetic research [9], ii) our hypothesis that the effect of genotype on stress may be driven by the Met-allele [6], [9] and iii) increasing the power of our sample to detect significant genotype differences. To increase insights into the data and utilize another measure reflecting neurotransmitter activity, BP_ND_ over the whole paradigm was also compared between genotype groups. BP_ND_ for each region of interest was used as a continuous dependent variable in the analyses and COMT genotype as categorical independent variable (Val-homozygotes vs. Met-hetero- and homozygotes). To investigate the effect of COMT Val^158^Met on subjective stress, a multilevel regression analysis was performed using the average subjective stress responses for each condition [11] (stress, control; see section “Psychosocial stress challenge”) as the dependent variable and COMT genotype (Val-homozygotes vs. Met-hetero- and homozygotes) as independent variable.

All analyses were repeated using Val/Val, Val/Met and Met/Met genotype as separate categories to indicate that the direction of the effect was the same for Met-hetero- and homozygotes.

All analyses were corrected for age, gender, nicotine use and alcohol consumption, but were stable when analyzed without covariates. A Simes-Hochberg correction was used to correct for multiple comparisons. Significance tests were performed in STATA version 11.0 [33]. Interactions analyses were not attempted (group*COMT genotype = [^18^F]fallypride displacement), given the uneven and sometimes low distribution of COMT genotype among participants. To give an indication of the direction and assist interpretation of the data each genotype group is shown separately in all figures.

## Results

### Sample

The study sample consisted of 26 individuals. Sociodemographic variables were not different for each COMT Val^158^Met genotype (Table 1) or for controls and first-degree relatives of psychosis patients (Table S1). The distribution of COMT Val^158^Met genotype was 30.8% (n = 8) Val/Val, 53.8% (n = 14) Val/Met, 15.4% (n = 4) Met/Met, and in Hardy-Weinberg equilibrium (χ^2^ (1) = .28, p = .59) (for distribution of COMT genotype across groups: Table 1 or Table S1).

**Table 1:** Sociodemographic variables for each COMT Val^158^Met genotype.

	Val/Val	Val/Met	Met/Met	Test statistic	P
**Age (SD)**	39 (14.1)	41.1 (15.6)	41 (17.7)	−44.7^1^	.15
**Gender, n (%)**				.17^2^	.92
male	5 (62.5%)	8 (57.1%)	2 (50%)		
**Education level, n (%)**				1.1^2^	.89
secondary education	1 (12.5%)	2 (14.3%)	1 (25%)		
bachelor degree	4 (50%)	9 (64.3%)	2 (50%)		
master degree	3 (37.5%)	3 (21.4%)	1 (25%)		
**Work situation, n (%)**				7.96^2^	.63
household	0	1 (7.14%)	1 (25%)		
school/education	0	3 (21.43%)	1 (25%)		
full-time employment	6 (75%)	5 (35.71%)	2 (50%)		
part-time employment	1 (12.5%)	3 (21.43%)	0		
self-employment	1 (12.5%)	2 (14.29%)	0		
**Marital status, n (%)**				1.7^2^	.78
married or cohabitating	5 (62.5%)	8 (57.2%)	2 (50%)		
divorced	0	1 (7.1%)	0		
never married	3 (37.5%)	5 (35.7%)	2 (50%)		
**GAF-score** 3 **(SD)**					
symptoms	82.3 (9.5)	79.6 (14.2)	90.5 (4.2)	.72^1^	.48
handicap	83.6 (9.9)	85.4 (6.8)	91.3 (4.8)	1.53^1^	.14
**Nicotine use (cigs/day), n (%)**					
0	6 (75%)	12 (78.6%)	3 (75%)		
0–10	1 (12.5%)	1 (7.1%)	0		
10< (max. 20)	1 (12.5%)	2 (14.3%)	1 (25%)		
**Alcohol cons. (grams/week), n (%)**				.81^1^	.43
0–50	6	8			
50–100	1	2			
100–150	4	4			
**Group (control/relative), n (%**)				3.94^2^	.14
control	5 (62.5%)	4 (28.57%)	3 (75%)		
first-degree relative	3 (37.5%)	10 (71.43%)	1 (25%)		
**[^18^F]fallypride parameters**					
injected activity (MBq)	181.60 (6.48)	195.80 (40.31)	185.62 (12.01)	.48^1^	.64
specific activity at injection time (GBq/µmol)	121.20 (60.59)	107.64 (63.57)	120.72 (32.60)	−.15^1^	.88
mass of unlabeled tracer injected (µg)	0.68 (0.32)	1.03 (0.77)	0.60 (0.22)	−.3^1^	.77

1 t-value.

2 Χ^2^-value.

3 GAF = Global assessment of functioning.

### Association between Task-induced [^18^F]fallypride Displacement and COMT Val^158^Met Genotype

**Figure 2:**
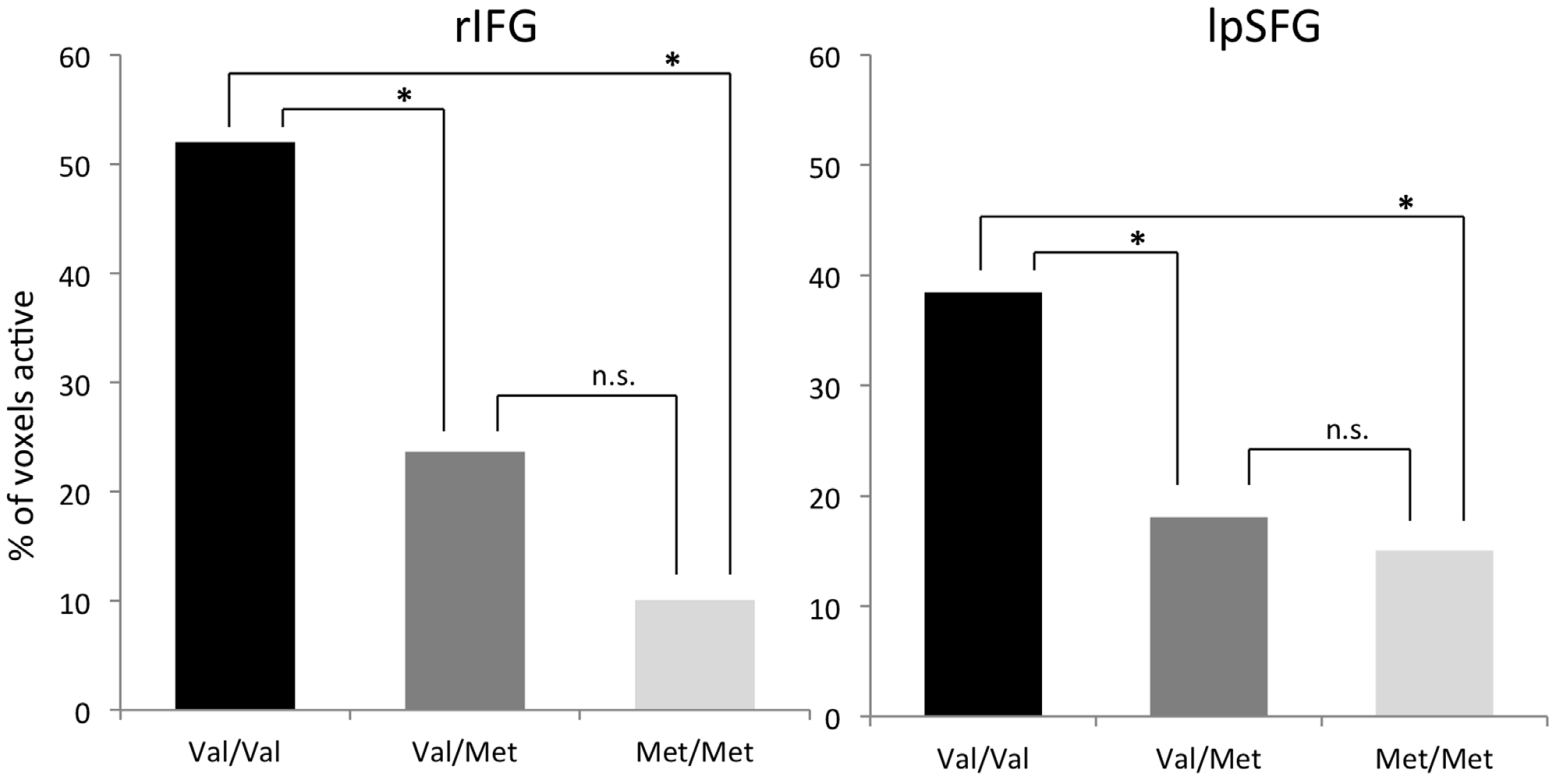
Genotype-dependent [^18^F]fallypride displacement in response to psychosocial stress. Comparison of the spatial extent to which [^18^F]fallypride displacement could be observed in VOIs, using parametric maps. Val-homozygotes showed the greatest difference in % of voxels active in response to stress per VOI (reflecting increased [^18^F]fallypride displacement), followed by Met-hetero- and homozygotes. The % of voxels active per VOI was not significantly different for Met-hetero- and homozygotes. Depicted numbers represent % of voxels active associated with stress (condition). Val/Met and Met/Met genotype are depicted separately to visualize the direction of the effect, but were grouped for the analyses. * = Exceeding threshold of p(corrected)<.05; n.s. = not significant. Note that the depicted % indicate the spatial extent to which ligand displacement was detected yet the % do not indicate VOI activity as a whole.

**Figure 3:**
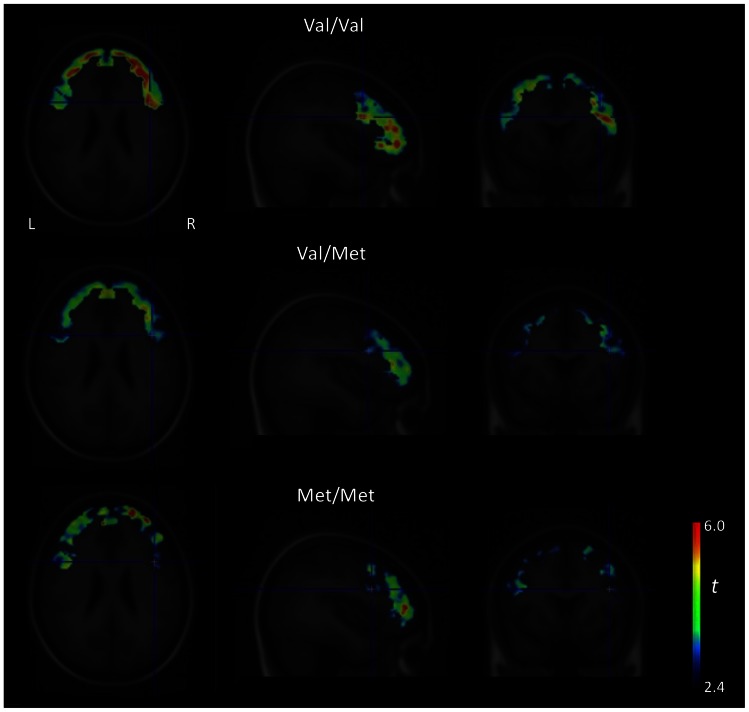
Mean t-maps per genotype group (Val/Val, Val/Met, Met/Met) reflecting [^18^F]fallypride displacement in response to psychosocial stress. Mean t-maps per genotype group (Val/Val, Val/Met, Met/Met) in transversal (left), sagittal (middle) and coronal (right) sections showing the spatial extent of [^18^F]fallypride displacement throughout the right inferior frontal gyrus (rlFG, BA 44) in response to the psychological stress task. Individual maps of t (with a cutoff of t>4.5, reflecting a one-sided t-test investigating increased ligand displacement versus the control state) were averaged for Val/Val (top row), Val/Met (middle row and Met/Met (bottom row) and illustrate the spatial extent of task-induced ligand displacement. Individual t-maps were generated using γ (*t* = γ/sd(γ) and averaged across all subjects for each genotype. The mean t-maps were overlaid on a T1-weighted MRI template. To visualize the direction of the effect, Met-hetero- and homozygotes were depicted separately, but were grouped together for the analyses. Images are thresholded for visualization purposes and were generated using PMOD v3.1.

A main effect of the task, the ability of the task to elicit changes in dopaminergic activity following psychosocial stress, has been described previously [11], [12]. Additionally, task-induced tracer-displacement was positively correlated with the subjective stress response [11], [21], internally validating the task. Results remained stable when group (control, first-degree relative) was entered as a covariate in the main effect analyses. COMT Val^158^Met was significantly associated with task-induced [^18^F]fallypride displacement in the left posterior frontal gyrus (lpSFG, BA8) and right inferior frontal gyrus, pars opercularis (rIFG, BA 44) (Table 2) (Fig. 2, Fig. 3), Val-homozygotes showing more [^18^F]fallypride displacement than Met-hetero- and homozygotes. Val-homozygotes and Met-allele carriers did not significantly differ in overall BP_ND_ (statistical parameters in table 2). Mean BP_ND_ values for Val-homozygotes and Met-allele carriers in the lpSFG and rIFG were.2 (sd = .08) vs.16 (sd = .16) and.26 (sd = .08) vs.21 (sd = .14) respectively. The percentage of voxels per VOI in which [^18^F]fallypride displacement in response to psychosocial stress could be detected is mentioned in table 3. Results did not change when Val/Val, Val/Met and Met/Met genotype were used as separate categories in the analyses (overall effect (COMT genotype as continuous variable): lpSFG: B = −.13, T = −3.01, 95% CI = −.21–−.04, p<.01; rIFG: B = −.22, T = −4.75, 95% CI = −.31–−.12, p = <.01). Met-hetero- and homozygotes did not significantly differ in terms of [^18^F]fallypride displacement (lpSFG: p = .65; rIFG: p = .09). To ensure that the results were not driven by an uneven count of controls and first-degree relatives in the genotype groups, we repeated the analysis using only controls/first-degree relatives. In both groups, Val-homozygotes showed more [^18^F]fallypride displacement than Met-hetero- and homozygotes (table 4). Finally, age was not significantly correlated with [^18^F]fallypride displacement (lpSFG; r = −.27, p = .18; rIFG: r = −.17, p = .4), yet revealed weak trends of older age being correlated with decreased stress-induced [^18^F]fallypride displacement.

**Table 2:** Main effect: Val-homozygotes demonstrated increased [^18^F]fallypride displacement to psychosocial stress compared to Met-hetero- and homozygotes (Val/Met - Met/Met).

Volume of Interest	B	95% CI	T	P
Ar Inferior Frontal Gyrus	−.29	−.44–−.15	−4.24	<.01*
Al posterior Superior Frontal Gyrus	−.2	−.32–−.08	−3.4	<.01*
Br Inferior Frontal Gyrus BP_ND_	−.05	−.17 −.08	−.8	.44
Bl posterior Superior Frontal Gyrus BP_ND_	−.04	−.18 −.09	−.7	.5
**Volume of Interest**	**B**	**95% CI**	**T**	**P**
Ar Inferior Frontal Gyrus	−.29	−.44–−.15	−4.24	<.01*
Al posterior Superior Frontal Gyrus	−.2	−.32–−.08	−3.4	<.01*
Br Inferior Frontal Gyrus BP_ND_	−.05	−.17 −.08	−.8	.44
Bl posterior Superior Frontal Gyrus BP_ND_	−.04	−.18 −.09	−.7	.5

Coding: Val/Val = [0] (reference group), Val/Met and Met/Met = [1].

A Represents the proportion of voxels in a given VOI in which significant stress-induced [^18^F]fallypride displacement could be detected using (γ (t = γ/sd(γ)) and threshold t>4.5.

B Overall binding potential relative to non-displaceable radioligand (BP_ND_).

* = Exceeding threshold of p(corrected)<.05 (p/16).

**Table 3:** Percentage of significant voxels displaying stress-induced [^18^F]fallypride displacement for Val-homozygotes and Met-allele carriers.

		Spatial extent of significant stress-induced [^18^F]fallypride displacement	
Volume of Interest	COMT genotype	Mean n (%)	SD
Ar Inferior Frontal Gyrus	Val-homozygotes	52%	18%
Ar Inferior Frontal Gyrus	Met-allele carriers	21%	15%
Al posterior Superior Frontal Gyrus	Val-homozygotes	38%	19%
Al posterior Superior Frontal Gyrus	Met-allele carriers	17%	12%

A Represents the percentage of voxels in a given VOI in which significant stress-induced [^18^F]fallypride displacement could be detected using (γ (t = γ/sd(γ) averaged across subjects per genotype and threshold t>4.5.

**Table 4:** Main effect analyses for controls and first-degree relatives separately: in both groups Val-homozygotes showed more stress-induced [^18^F]fallypride displacement than Met-hetero- and homozygotes.

Volume of Interest	Group	B	95% CI	T	P
r Inferior Frontal Gyrus	Controls	−.22	−.46–−.03	−2.18	.07
r Inferior Frontal Gyrus	Relatives	−.25	−.48–−.02	−2.46	.04
l posterior Superior Frontal Gyrus	Controls	−.37	−.74 −.01	−2.39	.05
l posterior Superior Frontal Gyrus	Relatives	−.3	−.58–−.02	−2.43	.04

Coding: Val/Val = [0] (reference group), Val/Met and Met/Met = [1].

### Association between Task-induced Subjective Stress Responses and COMT Val^158^Met Genotype

**Figure 4:**
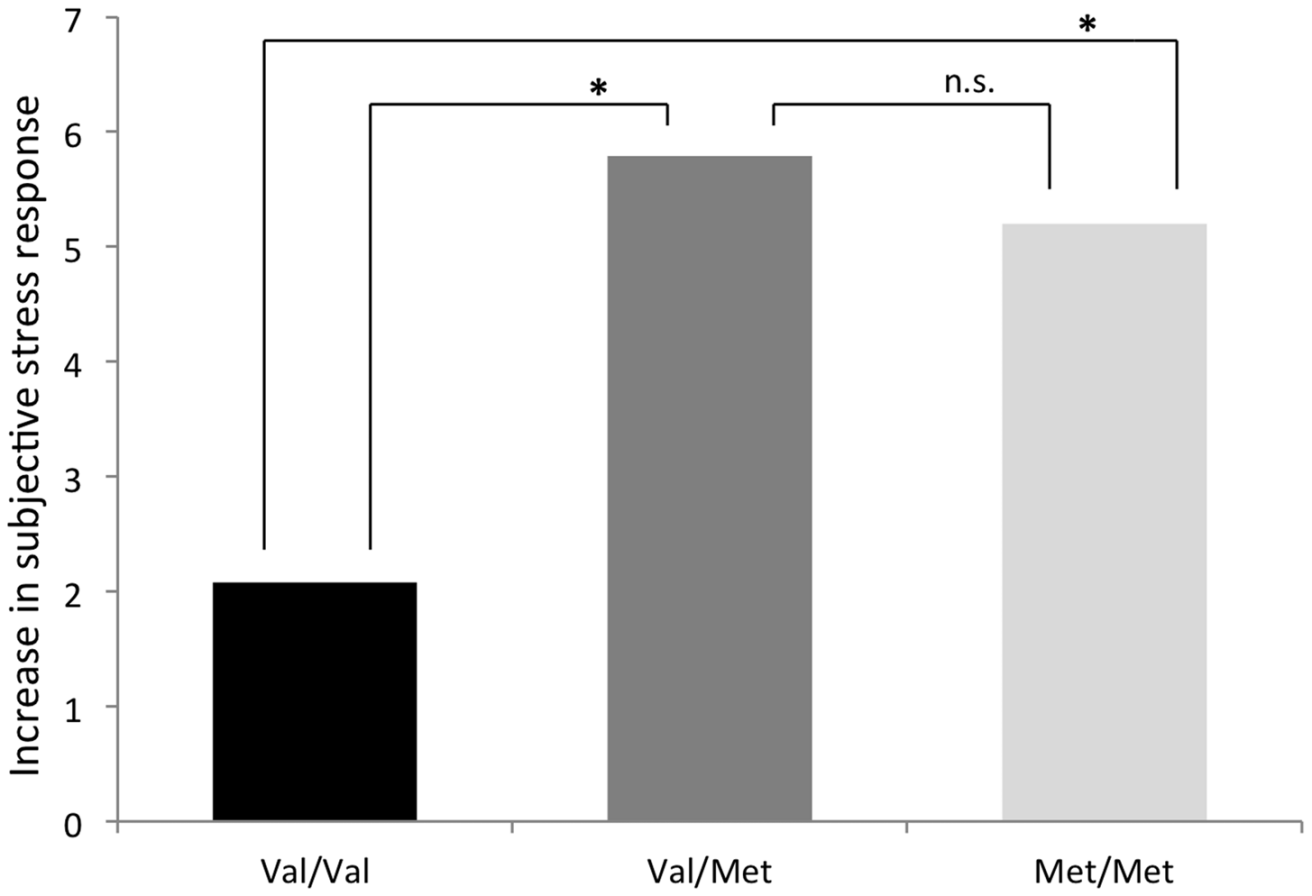
Genotype-dependent increases in the subjective stress response. There was a significant effect of COMT genotype on subjective stress responses, Val-homozygotes less reactive to stress than Met-hetero- and homozygotes. Increases in subjective stress responses were not significantly different for Met-hetero- and homozygotes. To visualize the direction of the effect, Met-hetero- and homozygotes were depicted separately, but were grouped together for the analyses. * = Exceeding threshold of p(corrected)<.05; n.s. = not significant.

COMT Val^158^Met was also associated with an increase in subjective stress from control to stress condition (B = 2.47, Z = 2.33, 95% CI = .39–4.55, p = .02), Val-homozygotes showing a smaller increase in stress response (mean change = 6.03, mean sd = 4.35) than Met-hetero- and homozygotes (mean change = 8.85, mean sd = 6.02) (Fig. 4). A similar direction was observed when Val/Val, Val/Met and Met/Met genotypes were used as separate categories (overall effect: B = 1.16, Z = 1.51, 95% CI = −.34–2.66, p = .13). Met-hetero- and homozygotes did not differ in increases in their subjective stress response (B = −1.14, Z = −.46, 95% CI = −6.04–3.75, p = .65). The direction of the effect of COMT genotype on subjective stress responses remained the same when the data was analyzed for controls and first-degree relatives separately (controls: B = .53, Z = .39, 95% CI = −2.18–3.24, p = .7; relatives: B = 3.87, Z, = 2.28, 95% CI = .55–7.19, p = .02). Age was not significantly correlated with subjective feelings of stress (mean stress in experimental condition – mean stress in control condition) (r = ,−.01 p = .99).

## Discussion

### COMT-dependent Regulation of PFC Dopamine and Human Stress Response

The results presented in the current manuscript suggest that subtle changes in PFC [^18^F]fallypride displacement following a stress challenge are dependent on COMT Val^158^Met genotype, Val-homozygotes showing larger displacement of [^18^F]fallypride, indicative of increased dopamine signaling or reduced dopamine degradation, than Met-hetero- homozygotes. Additional analyses seem to hint at the idea that there is a dose-response association between COMT genotype and stress-induced [^18^F]fallypride displacement, Val-homozygotes showing the most stress-induced [^18^F]fallypride displacement, followed by Met-heterozygotes and Met-homozygotes. On a behavioral level, subjective stress responses to the task were smaller in Val-homozygotes than Met-hetero- and homozygotes, in line with other behavioral observations [8], [9]. The similarities in outcome measures between Met-hetero- and homozygotes may indicate that molecular mechanisms that regulate dopaminergic processing may be more alike in Met-hetero- and homozygotes than in Val-homozygotes and Met-heterozygotes [9], although should be interpreted with caution due to the nature of the subtle effects and a modest sample size. Interestingly, one such mechanism, Val-allele methylation, has been shown to crucially influence dopamine-mediated PFC activity in Val-homozygotes, but did not predict brain activity in Met-heterozygotes [34]. Observations of decreased PFC [^18^F]fallypride displacement in response to stress may indicate that carriers of the Met-allele may be more vulnerable to the adverse effects of stress than Val-homozygotes, which is in line with a recent behavioral study showing Val-specific stress-resistance during a working memory task [35].

A number of studies to date have reported stress-induced increases in subcortical dopaminergic transmission, following experimental lowering of PFC dopamine [36], [37]. These reports suggest that prefrontal dopaminergic activity regulates the release of subcortical dopamine and make it attractive to hypothesize that the observed increase in feelings of stress in Met-carriers were generated by an increase in subcortical dopamine release. Slight differences in Met-specific attenuation of frontal dopaminergic activity in areas such as the IFG and SFG under stress may initiate a cascade of events, leading to increased mesolimbic dopamine release. In turn, intricate connections between mesolimbic dopaminergic nuclei (e.g. striatum) and stress-hormone (e.g. cortisol) producing systems such as the hypothalamic-pituitary-adrenal (HPA) axis may underlie increased stress-sensitivity in Met-allele carriers, but not in Val-homozygotes. This mechanism seems to fit with observations of Met-allele carriers displaying a more sensitive biological stress response (e.g. cortisol) than Val-homozygotes [9], [38] yet remains a speculative framework.

Additionally, our findings seemingly agree with the warrior/worrier hypothesis [6], which states that the selective effects of COMT may make Met-allele carriers more stress-sensitive, yet enhance cognitive performance. Although the aim of the present study was not to confirm the latter observation, evidence for subtle cognitive benefits of Met-allele carriers over Val-homozygotes is extensive [3], [17] and our data add to the suggestion that Met-allele carriers are behaviorally most sensitive to psychosocial stressors.

### COMT-dependent Dopaminergic Activity in the PFC

COMT-dependent changes in [^18^F]fallypride displacement were observed in the left posterior frontal gyrus (pSFG) and right inferior frontal gyrus (IFG), areas which have been associated with genotype-dependent (dopaminergic) PFC activity [5], [39] during emotional and working memory paradigms. Although in vivo estimations of cortical dopamine release in humans are scarce, our observation that in vivo measurements of PFC dopamine release are COMT-dependent is in line with a report by Stokes et al. [39], who observed COMT-dependent middle frontal gyrus (MFG) and SFG dopamine release in response to Δ^9^-tetrahydrocannabinol, with respectively Val-homozygotes and Met-homozygotes showing the largest and smallest decrease in [^11^C]raclopride BP_ND_. Although the present study used a different radioligand, the current results agree with those of findings of Stokes et al. [39] as they demonstrate COMT-dependent dopaminergic activity in cortical areas. Furthermore, a large body of fMRI studies suggest COMT-dependent task-related PFC activity, in among others the IFG [40] and SFG [3], [17], with the direction of the allele effect dependent on task demands (cognitive or emotional) [5]. Thus, evidence suggests that the effect of COMT on task-related (dopaminergic) activity can be detected robustly in PFC cortical regions, task-dependent allelic effects indicating that brain activity during cognitive and emotional stress paradigms is different for Val and Met-carriers.

As to where the observed lateralization of dopaminergic activity in the rIFG and lpSFG stems from, it remains speculative. As mentioned above, dopaminergic activity in the lSFG has previously been reported to be COMT-dependent [39]. In addition, findings of an fMRI study by Yacubian and colleagues [41] revealed that activity in the rIFG, or more general right dorsolateral PFC activity, during an emotional (reward) task is COMT-dependent, hinting at dopaminergic activity. Such findings may indicate that areas such as lpSFG and rIFG may be part of a lateralized dopaminergic network, with its workings heavily influenced by the COMT genotype. Given the scarcity of studies that have investigated COMT-dependent dopaminergic activity in the PFC, dopaminergic lateralization in this area of the human brain is a phenomenon that should be further looked into.

### The Role of PFC Dopamine in Psychosis

The interpretation of results presented in this manuscript may be of potential use for stress-related psychiatric disorders such as psychosis. Psychotic complaints have been associated with abnormally high subcortical dopaminergic activity [42] and may be associated with low levels of PFC dopamine [16], suggesting an imbalance between these two dopaminergic regions [16], [43]. Additionally, a subcortical hyperdopaminergic response to stress can be observed in prodromal psychosis [44], while decreased levels of PFC dopamine may reflect decreased functionality of the PFC to prevent a subcortical hyperdopaminergic state [16]. Our observations of COMT-dependent stress-induced [^18^F]fallypride displacement in the human PFC suggest that the integrity of this “brake-like” PFC function may be genotype-dependent.

### Implications for Theoretical Frameworks of Dopaminergic Functioning

Theoretical models of dopaminergic functioning such as the inverted-U curve describe cognitive performance as optimal at an intermediate level of PFC dopaminergic activity but not at the lowest or highest ends of the curve [45], [46]. Our results seemingly suggest that the inverted-U relation between stress and PFC dopaminergic functioning is COMT-dependent: PFC dopamine levels in Val-homozygotes, who may have low baseline levels of PFC dopamine [1], [2], [4], [17] due to higher enzymatic activity, increased substantially after stress as demonstrated by the large increase in [^18^F]fallypride displacement. Met-allele carriers, hypothesized to have higher PFC dopaminergic tone [4] due to lower enzymatic activity, showed a small increase, which may be related to their baseline dopamine levels being close to their peak dopamine levels (i.e. a ceiling effect) [1], [3]. Genotype-specific changes in dopamine release or degradation following stress may have increased PFC dopamine levels of Val-homozygotes closer to their optimum, at which stress-resistance may be enhanced, while pushing Met-carriers over their optimum, making them susceptible to the adverse effects of stress.

Given the use of the current design, a) a task with a control condition and a stress condition (no rest/basal conditions) and b) the use of the LSRRM, which renders voxel-wise comparisons of tracer displacement in response to a stimulus (difference score), we were unfortunately not able to directly test this hypothesis. However, although the framework we propose remains speculative, low dopaminergic cortical tone during rest Val-homozygotes has already been observed [4] and it is supported by studies showing COMT-dependent brain activity [5], [47] and behavior [35]. Moreover, our interpretation is in essence similar to the framework proposed by Mattay et al [3], who observed a COMT-dependent association between PFC efficiency (signal-to-noise ratio), an indication of PFC dopaminergic activity [48], and cognition following amphetamine administration.

### Strengths and Limitations

To the best of our knowledge, the current study is the first to report an effect of the COMT genotype on stress-induced changes in the spatial extent of PFC [^18^F]fallypride displacement and subjective stress responses in humans, augmenting other reports on the key role of COMT in stress-related behavior and providing speculation for its underlying neurocircuitry. First and foremost it should be pointed out that our modest sample size may have limited our power to detect or obscured subtle effects in addition to the effects reported in this manuscript. Additionally, the mixed sample of healthy controls and healthy first-degree relatives could have influenced the reported outcome measures. Although we attempted to control for group differences by including group as a covariate and analyzing the results for each group separately, our statistical model may have not accounted for all of the group-specific differences. A recent study by Lataster et al [21], using the same sample, reported between-group differences in other dopaminergic PFC areas, hinting at complex interactions between group and genotype which may be difficult to disentangle and could have influenced the reported outcome measures.

A lack of additional genes that may have influenced dopaminergic activity, such as DAT and MTHFR [49], may have provided a less complete overview of the effects of COMT on subjective stress and its underlying neurocircuitry. Therefore future replications using a large sample size, more genetic variation associated with dopaminergic function and perhaps a patient group may further reveal and refine between-group similarities and differences.

An association between age and [^18^F]fallypride displacement/behavioral stress-sensitivity was not observed in the current study. Associations between dopaminergic activity and age have been reported previously and seem to affect both dopamine D1 [50] and D2 receptor activity [51], [52]. The absence of an effect of age in the current manuscript may have affected our outcome measures. However, given that the distribution of age did not differ among COMT genotype or group (control/first-degree relative), we expect such effects to be rather small. In addition, the absence of an effect of age on our outcome measures may also indicate that stress-related dopaminergic activity displays a certain robustness to advancing age, whereas cognitive performance and associated dopaminergic processing may decline with advancing age [46], [50], [51], [52].

PET measurement of alterations in dopamine concentration in response to a pharmacological manipulation or during a behavioral task can be obtained by calculating the percentage change in dopamine D_2/3_ receptor BP_ND_ (ΔBP_ND_), measured under dual scanning conditions (control and activation condition) [53], [54]. This design has the advantage that the quantitative index of dopamine release, ΔBP_ND_, is obtained by applying standard techniques such as the simplified reference tissue model (SSRM) [55]. However BP_ND_ measurement in the activated condition assumes that the subject is in steady state during activation. In addition, the need for two separate BP_ND_ measurements and possibly noisy subtraction of two low BP_ND_ values in extrastriatal regions could reduce the sensitivity of the design [23] and may partially account for the mixed results of studies investigating amphetamine-induced ΔBP_ND_ changes in frontal cortical regions [53], [54], [56]. Simulations demonstrated also that ΔBP_ND_ has an inherent sensitivity to timing of dopamine perturbations and could lead to incorrect inferences of the relative amounts of dopamine released during conditions [57]. In the present study, we implemented a common variant of the SSRM, the LSRRM, which has several practical advantages, such a single scanning session to avoid possible session effects. Fundamentally, it is based on a kinetic model of the stimulus-induced physiological phenomenon involved, where non-steady-state effects are considered by making parameters time-dependent. The presence of significant dopamine-induced transient changes in ligand displacement after the stimulus initiation is estimated by fitting the model to data from individual subjects, therefore facilitating the detection of relatively small differences in dopamine release, which is of particular interest for areas with low signal-to-noise ratio such as the PFC. Moreover, since the model allows for voxel-wise parametric calculations of the time-dependent parameters, it allows direct comparisons of spatial extent of dopamine release between subject populations within a specific region of interest [57].

On the other hand, in the LSRRM approach, possible alterations in regional cerebral blood flow (rCBF) are not fully accounted for. However, as shown by previous simulation studies [23], [25], it is unlikely that rCBF-related changes would add major perturbations in ligand displacement using a behavioural activation paradigm. Second, the current experimental design did not allow to investigate striatal dopamine release simultaneously, as task timing is dependent on regional D_2/3_ density [26] and high D_2/3_ receptor density in the striatal regions require a longer baseline scan duration (2–3 hours) in order to reach a similar proportion of receptors to be occupied by the dopamine-competing [^18^F]fallypride ligand, hence sensitivity for detection, while a postponed task initiation of at least 190 minutes post-injection is needed to evaluate both [26], [31]. Scan duration could therefore be of potential interest for future studies implementing the LSRRM. Finally, in the current manuscript we investigated the spatial extent of ligand displacement, rather than ΔBP_ND_. The spatial extent of ligand displacement reflects the percentage of voxels in which [^18^F]fallypride displacement can be detected, and although a strong correlation with BP_ND_ exists [27], may be less straightforwardly interpreted than other standard measure of dopamine release such as BP_ND_.

## Supporting Information

Table S1 **Demographics for healthy controls and healthy individuals at increased risk of psychosis.** ^1^t-value. ^2^Χ^2^-value. ^3^GAF = Global assessment of functioning.
(DOCX)

Text S1 **Radiotracer preparation.**
(DOCX)

Text S2 **The kinetic model.**
(DOCX)
