# Supplementary material for: COMT Val158Met Genotype Selectively Alters Prefrontal [18F]Fallypride Displacement and Subjective Feelings of Stress in Response to a Psychosocial Stress Challenge
Source: PLoS One. 2013 Jun 14;8(6):e65662. doi: 10.1371/journal.pone.0065662 (PMC3683024; doi:10.1371/journal.pone.0065662)
Supplement: Table S1 — Demographics for healthy controls and healthy individuals at increased risk of psychosis. 1t-value. 2Χ2-value. 3GAF = Global assessment of functioning. (DOCX) [file pone.0065662.s001.docx]

**Table S3 Demographics for healthy controls and healthy individuals at increased risk of psychosis.**

|  | **Controls (n=12)** | **Relatives (n=14)** | **Test statistic** | **P** |
| --- | --- | --- | --- | --- |
| **Age (SD)** | 39.75 (15.67) | 41 (14.62) | .21^1^ | .84 |
| **Gender, n (%)** |  |  | .74^2^ | .39 |
| male | 8 (66.67%) | 7 (50%) |  |  |
| **Education level, n (%)** |  |  | 4.06^2^ | .4 |
| secondary education | 1 (8.33%) | 3 (21.4%) |  |  |
| bachelor degree | 6 (50.17%) | 7 (50%) |  |  |
| master degree | 5 (41.5%) | 4 (28.4%) |  |  |
| **Work situation, n (%)** |  |  | 7.82^2^ | .17 |
| household | 1 (8.33%) | 1 (7.14%) |  |  |
| school/education | 0 | 4 (28.57%) |  |  |
| full-time employment | 9 (75%) | 4 (28.57%) |  |  |
| part-time employment | 1 (8.33%) | 3 (21.43%) |  |  |
| self-employment | 1 (8.33%) | 2 (14.29%) |  |  |
| **Marital status, n (%)** |  |  | 2.69^2^ | .44 |
| married or cohabitating | 6 (50%) | 8 (57.14%) |  |  |
| divorced | 2 (16.66%) | 0 |  |  |
| never married | 4 (33.34%) | 6 (42.86%) |  |  |
| **GAF-score^3^ (SD)** |  |  |  |  |
| symptoms | 84.58 (7.53) | 81.88 (9.61) | -.71^1^ | .49 |
| handicap | 84.17 (7.64) | 84.88 (8.95) | .19^1^ | .85 |
| **Nicotine use (cigs/day), n (%)** |  |  |  |  |
| 0 | 9 (75%) | 11 (78.57%) |  |  |
| 0 - 10 | 2 (16.66%) | 1 (7.14%) |  |  |
| 10< (max. 20) | 1 (8.33%) | 2 (14.28%) |  |  |
| **Alcohol cons. (grams/week), n (%)** |  |  | -.51^1^ | .62 |
| 0 - 50 | 6 (50%) | 8 (57.14%) |  |  |
| 50 - 100 | 2 (16.66%) | 2 (14.29%) |  |  |
| 100 - 150 | 4 (33.34%) | 4 (28.57%) |  |  |
| **COMT Val158Met genotype, n (%)** |  |  | 3.94^2^ | .14 |
| Val/Val | 5 (41.67%) | 3 (21.43%) |  |  |
| Val/Met | 4 (33.33%) | 10 (71.43%) |  |  |
| Met/Met | 3 (25%) | 1 (7.14%) |  |  |
| **[^18^F]Fallypride parameters (SD)** |  |  |  |  |
| injected activity (MBq) | 197.53 (43.70) | 183.30 (7.08) | -1.21^1^ | .24 |
| specific activity at injection time (GBq/μmol) | 118.33 (62.68) | 110.14 (53.90) | -.35^1^ | .73 |
| mass of unlabeled tracer injected (μg) | 0.80 (0.49) | 0.89 (0.72) | -.03^1^ | .98 |

**Supporting information legend**

^1^t-value
^2^Χ^2^-value
^3^GAF = Global assessment of functioning
