# Supplementary material for: COMT Val158Met Genotype Selectively Alters Prefrontal [18F]Fallypride Displacement and Subjective Feelings of Stress in Response to a Psychosocial Stress Challenge
Source: PLoS One. 2013 Jun 14;8(6):e65662. doi: 10.1371/journal.pone.0065662 (PMC3683024; doi:10.1371/journal.pone.0065662)
Supplement: Text S1 — Radiotracer preparation. (DOCX) [file pone.0065662.s002.docx]

**S1 Radiotracer preparation**

The fluorinated substituted benzamide [^18^F]fallypride is a high affinity dopamine D_2/3_ receptor antagonist radiotracer previously used to visualize and estimate both striatal and extrastriatal dopamine levels [[1](#_ENREF_1),[2](#_ENREF_2),[3](#_ENREF_3)]. The [^18^F]fallypride was produced according to following reported method. The precursor for tracer synthesis was obtained from ABX (Radeberg, Germany) and labeling was performed on-site using a Raytest Synchrom R&D synthesis module (Raytest, Straubenhardt, Germany). Water, enriched in oxygen-18 (90-99% enrichment) and contained in a closed niobium target was irradiated with 18-MeV protons accelerated in a cyclotron to provide [^18^F]fluoride (fluorine-18 has a half-life of 109.8 min) by the ^18^O(p,n)^18^F nuclear reaction. Possible undesired nuclear side reactions included ^16^O(p,α)^13^N and ^16^O(p,pn)^15^O yielding small amounts (<5%) of short lived nitrogen-13 (half-life 10 min) and oxygen-15 (half-life 2 min) at the end of the irradiation. As the synthesis sequence takes approximately 60 minutes, the amount of these short lived radionuclidic impurities was reduced to <500 ppm by the decay. The aqueous solution of [^18^F]fluoride was transferred to an automated synthesis module where the [^18^F]fluoride was extracted from the solution using an anion exchange column. The [^18^F]fluoride was then desorbed from the anion exchange column with a solution of K_2_CO_3_/Kryptofix and the resulting solution was evaporated. The residue was dissolved in anhydrous acetonitrile and the solution was again evaporated. A solution of (S)-2,3-dimethoxy-5-[3-[[(4-methylphenyl)-sulfonyl]oxy]-propyl)-*N*-[[1-(2-propenyl)-2-pyrrolidinyl]methyl]-benzamide in anhydrous DMF was added to the residue and the mixture was heated to provide the crude [^18^F]fallypride. After cooling, the mixture was diluted with aqua ad injectabilia. Purification of the crude [^18^F]fallypride was performed by reverse-phase high performance liquid chromatographic (RP-HPLC) using a Waters XTerra^TM^ RP18 5 µm 7.8 mm x 150 mm column and sodium acetate (NaOAc) 0.05M pH5.5 / Ethanol 70:30 V/V as mobile phase at a flow rate of 1.5 ml/min. The purified [^18^F]fallypride solution was diluted with NaCl 0.9 %. Due to the short half-life of fluorine-18 (109.8 min), this solution was immediately filtered through a Millipore Cathivex-GS 0.22 µm filter to yield a final, sterile solution of [^18^F]fallypride (drug product). The final product of the radioligand was administered as a sterile solution of 7 mM sodium acetate buffer pH 5.5, 0.72% NaCl and 6% ethanol. Characterization and quality control were performed on the drug product. Process controls included continuous monitoring of radioactivity, reaction temperatures and reactor pressure and visual verification of addition of reagents to the reactor (the radiochemical purity was > 95%). The specific activity at the time of injection was ≥ 40 GBq/μmol (range 41 - 238 GBq/μmol) and the amount of tracer injected was on average 183 MBq (range 167 - 333 MBq) (see table 1 and S3).

**References**

1. Ceccarini J, Vrieze E, Koole M, Muylle T, Bormans G, et al. (2012) Optimized in vivo detection of dopamine release using 18F-fallypride PET. Journal of nuclear medicine : official publication, Society of Nuclear Medicine 53: 1565-1572.

2. Christian BT, Lehrer DS, Shi B, Narayanan TK, Strohmeyer PS, et al. (2006) Measuring dopamine neuromodulation in the thalamus: using [F-18]fallypride PET to study dopamine release during a spatial attention task. NeuroImage 31: 139-152.

3. Lataster J, Collip D, Ceccarini J, Haas D, Booij L, et al. (2011) Psychosocial stress is associated with in vivo dopamine release in human ventromedial prefrontal cortex: a positron emission tomography study using [(1)F]fallypride. NeuroImage 58: 1081-1089.
