# Supplementary material for: COMT Val158Met Genotype Selectively Alters Prefrontal [18F]Fallypride Displacement and Subjective Feelings of Stress in Response to a Psychosocial Stress Challenge
Source: PLoS One. 2013 Jun 14;8(6):e65662. doi: 10.1371/journal.pone.0065662 (PMC3683024; doi:10.1371/journal.pone.0065662)
Supplement: Text S2 — The kinetic model. (DOCX) [file pone.0065662.s003.docx]

**S2 The kinetic model**
PET emission was performed conform the one-day PET imaging protocol for [^18^F]fallypride described and used previously by Badgaiyan et al. [[1](#_ENREF_1)]. Emission data were collected in two segments, one during the control condition (86 minutes) and one during the stress condition of the MIST psychosocial stress task (100 minutes). Data were obtained in 60 s frames during the first 6 minutes and in 120 s frames thereafter. Given the use of an “activation” parameter in the kinetic model used for analyses [[2](#_ENREF_2)] (discussed below), representing presence or absence of additional dopamine release, and the hypothesis of stress being associated with increased dopaminergic activity, the stress condition of the MIST was always presented after the control condition. Conform previous work [[1](#_ENREF_1),[2](#_ENREF_2),[3](#_ENREF_3),[4](#_ENREF_4),[5](#_ENREF_5),[6](#_ENREF_6),[7](#_ENREF_7)], estimation of kinetic parameters was performed by applying the linearized simplified reference region model (LSRRM[[4](#_ENREF_4)]), an extension of the simplified reference region model (SRRM[[8](#_ENREF_8),[9](#_ENREF_9)]), modified to include time-dependent parameters and linearized for all estimated parameters [[10](#_ENREF_10)]. The LSRRM kinetic model is a well-documented mathematical method to investigate ligand displacement following experimental manipulation [[1](#_ENREF_1),[2](#_ENREF_2),[3](#_ENREF_3),[4](#_ENREF_4),[5](#_ENREF_5),[6](#_ENREF_6)]. As previously described [[1](#_ENREF_1),[2](#_ENREF_2),[3](#_ENREF_3),[4](#_ENREF_4)], the LSRRM takes into account temporal perturbations in ligand specific binding by assuming that the steady physiological state is not maintained, making it suitable for assessing task-related ligand displacement in an experimental design that involves a change of task condition from control to a dopamine activation paradigm during one single scan session. For a detailed description of the functional time-dependent equations describing the *in vivo* kinetics of radioligand please see the work of Ceccarini and colleagues [[3](#_ENREF_3)]. Briefly, the LSRRM allows the dissociation rate of ligand from the receptor, k_2a_, to change through the paradigm in response to fluctuating levels of dopamine (k_2a_=k_2_/[1+BP_ND_]), where k_2_ is the tissue to plasma efflux constant in the tissue region and BP_ND_ is the nondisplaceable binding potential [[11](#_ENREF_11)]. Changes in BP_ND_ in activation studies are usually assumed to reflect changes in the concentration of available neuroreceptor sites (B_avail_), and a decrease in BP_ND_ is assumed to reflect increased dopamine release. The temporal change of k_2a_ (via a change in BP_ND_) is obtained by introducing the additional term γ·h(t), where γ represents the amplitude of the ligand displacement and the function h(t) describes a rapid change following task onset and dissipation over time. The exponential decay function h(t)=exp[−τ(t−T)] accounts for temporal variation in the model parameters, where τ controls the rate at which activation effects die away (set to τ=0.03 min^-1^, conform previous work [[3](#_ENREF_3),[4](#_ENREF_4)]) and T indicates the task initiation time (T=100 minutes postinjection). It follows that, through linearization of the simplified reference region model (SRRM), an increased k_2a_ reflected in a decreased BP_ND_ for dopamine receptors, due to increased dopamine release, would result in a positive value of γ. The LSRRM model uses weighted linear least squares analysis for parameter estimation [[2](#_ENREF_2)].

**References**

1. Badgaiyan RD, Wack D (2011) Evidence of dopaminergic processing of executive inhibition. PloS one 6: e28075.

2. Alpert NM, Badgaiyan RD, Livni E, Fischman AJ (2003) A novel method for noninvasive detection of neuromodulatory changes in specific neurotransmitter systems. NeuroImage 19: 1049-1060.

3. Ceccarini J, Vrieze E, Koole M, Muylle T, Bormans G, et al. (2012) Optimized in vivo detection of dopamine release using 18F-fallypride PET. Journal of nuclear medicine : official publication, Society of Nuclear Medicine 53: 1565-1572.

4. Christian BT, Lehrer DS, Shi B, Narayanan TK, Strohmeyer PS, et al. (2006) Measuring dopamine neuromodulation in the thalamus: using [F-18]fallypride PET to study dopamine release during a spatial attention task. NeuroImage 31: 139-152.

5. Lataster J, Collip D, Ceccarini J, Haas D, Booij L, et al. (2011) Psychosocial stress is associated with in vivo dopamine release in human ventromedial prefrontal cortex: a positron emission tomography study using [(1)F]fallypride. NeuroImage 58: 1081-1089.

6. Vrieze E, Ceccarini J, Pizzagalli DA, Bormans G, Vandenbulcke M, et al. (2011) Measuring extrastriatal dopamine release during a reward learning task. Human brain mapping.

7. Backman L, Nyberg L, Soveri A, Johansson J, Andersson M, et al. (2011) Effects of working-memory training on striatal dopamine release. Science 333: 718.

8. Lammertsma AA, Bench CJ, Hume SP, Osman S, Gunn K, et al. (1996) Comparison of methods for analysis of clinical [11C]raclopride studies. Journal of cerebral blood flow and metabolism : official journal of the International Society of Cerebral Blood Flow and Metabolism 16: 42-52.

9. Gunn RN, Lammertsma AA, Hume SP, Cunningham VJ (1997) Parametric imaging of ligand-receptor binding in PET using a simplified reference region model. NeuroImage 6: 279-287.

10. Friston KJ, Malizia AL, Wilson S, Cunningham VJ, Jones T, et al. (1997) Analysis of dynamic radioligand displacement or "activation" studies. Journal of cerebral blood flow and metabolism : official journal of the International Society of Cerebral Blood Flow and Metabolism 17: 80-93.

11. Innis RB, Cunningham VJ, Delforge J, Fujita M, Gjedde A, et al. (2007) Consensus nomenclature for in vivo imaging of reversibly binding radioligands. Journal of cerebral blood flow and metabolism : official journal of the International Society of Cerebral Blood Flow and Metabolism 27: 1533-1539.
